# Advanced Proton-Conducting Ceramics Based on Layered Perovskite BaLaInO_4_ for Energy Conversion Technologies and Devices

**DOI:** 10.3390/ma15196841

**Published:** 2022-10-01

**Authors:** Nataliia Tarasova, Anzhelika Bedarkova

**Affiliations:** 1 Laboratory of Electrochemical Devices Based on Solid Oxide Proton Electrolytes, The Institute of High Temperature Electrochemistry of the Ural Branch of the Russian Academy of Sciences, 620660 Yekaterinburg, Russia; 2 Institute of Hydrogen Energy, Ural Federal University, 620002 Yekaterinburg, Russia

**Keywords:** solid oxide fuel cell, BaLaInO_4_, layered perovskite, Ruddlesden-Popper structure, proton conductivity

## Abstract

Production of high efficiency renewable energy source for sustainable global development is an important challenge for humans. Hydrogen energy systems are one of the key elements for the development of sustainable energy future. These systems are eco-friendly and include devices such as protonic ceramic fuel cells, which require advanced proton-conducting materials. In this study, we focused on new ceramics with significantly improved target properties for hydrogen energy purposes. Neodymium-doped phase based on layered perovskite BaLaInO_4_ was obtained for the first time. The ability for water intercalation and proton transport was proved. It was shown that the composition BaLa_0.9_Nd_0.1_InO_4_ is the predominant proton conductor below 400 °C under wet air. Moreover, isovalent doping of layered perovskites AA′BO_4_ is the promising method for improving transport properties and obtaining novel advanced proton-conducting ceramic materials.

## 1. Introduction

The goal of sustainable global development is an important challenge of human society for many decades [1,2,3,4,5,6,7,8]. The creation and production of high efficiency renewable energy source is a major opportunity for this achievement [9,10,11,12,13,14,15]. Hydrogen can be considered as one of the key elements for the development of sustainable energy future [16,17,18,19]. The eco-friendly hydrogen-production and hydrogen-operation systems include devices such as protonic ceramic fuel cells and protonic ceramic electrolysis cells, which require advanced proton-conducting materials [20,21,22,23,24,25,26,27,28,29,30,31,32]. Such type of solid-state conductors was discovered in early 1980s by Iwahara et al. and these phases were derivatives from strontium cerate [33,34,35]. These complex oxides are characterized by the perovskite structure, and many of recent developed protonic conductors have related structure, for example structure of double perovskites [36] or hexagonal perovskites [37,38].

At the same time, the development of hydrogen energy devices requires not only the creation of novel materials with improved properties but the solution of the problem of materials comparability with each other in electrochemical device also. Today, one of the promising cathode materials is layered perovskite based on Ln_2_NiO_4+δ_ [39,40,41,42]. Likely enough, the similarity of crystal structure can help to resolve the problem of comparability of electrode and electrolyte components. By this way, the materials search of advanced proton conductors characterized by the layered perovskite structures is on the focus.

**Figure 1:**
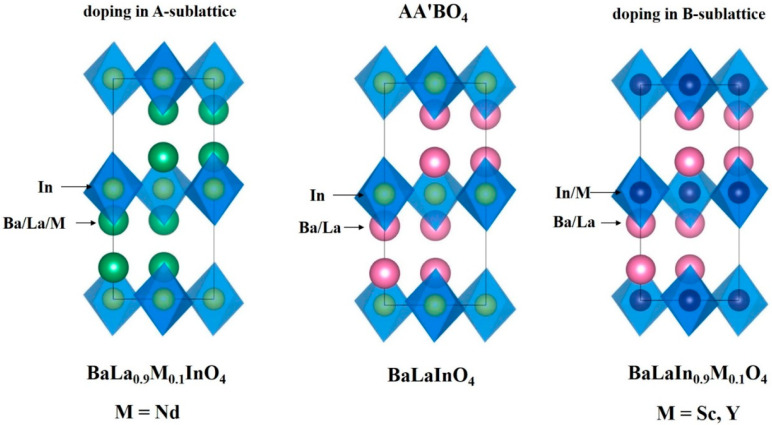
The isovalent substitution representation of BaLaInO_4_.

Layered perovskites with general formula AA′BO_4_ where A is Ba or Sr, A′ is La or Nd, and B is In or Sc were described as ionic (oxygen-ionic and protonic) conductors in the past few years [43,44,45,46,47,48,49,50,51,52,53,54,55,56,57,58,59,60,61,62]. It was proved that doping in the cationic sublattices is the successful way for improving protonic conductivity up to ~1.5 orders. At the same time, most of the works are related to the methods of heterovalent doping. Doping by the alkali-earth cations such as Ca^2+^, Sr^2+^, Ba^2+^ into La^3+^ (acceptor doping) [49] sublattice and Ti^4+^, Zr^4+^, Nb^4+^ into In^3+^ sublattice [50] (donor doping) was investigated. However, isovalent doping shows even more meaningful results. Earlier, we reported about the possibility of isovalent doping of B-sublattice of layered perovskite BaLaInO_4_ by the ions with different radii (Y, Sc) [54,55,56]. In this paper, we focused on the modification of BaLaInO_4_ structure by the isovalent doping in the A (lanthanum) sublattice (Figure 1). Neodymium was chosen as a trivalent metal with a close radius to lanthanum. The Nd-doped composition BaLa_0.9_Nd_0.1_InO_4_ was studied as the proton conductor for the first time. In addition, the comparative analysis with previously obtained data was carried out. The effect of dopant nature on the crystal lattice parameters and on the mobility of ions was revealed. 

## 2. Materials and Methods

Layered perovskite BaLa_0.9_Nd_0.1_InO_4_ was prepared by the solid-state method. The carbonates BaCO_3_, CaCO_3_ and oxides Nd_2_O_3_, In_2_O_3_ were used. 

The X-ray analysis was made using a Bruker Advance D8 diffractometer (Bruker, Billerica, MA, USA). Morphology of the powder sample was defined by Phenom ProX (ThermoFisher, Waltham, MA, USA) Desktop scanning electron microscope (SEM) integrated with energy-dispersive X-ray diffraction (EDS) detector.

The thermogravimetry (TG) analysis was made using STA 409 PC Netzsch Analyzer (Netzsch, Selb, Germany). The initially hydrated samples were used for the investigations. 

The electrical conductivity was measured using impedance spectrometer Z-1000P (Elins, RF). The samples were cooled from 1000 to 200 °C under dry (*p*H_2_O = 3.5 × 10^−5^ atm) or wet (*p*H_2_O = 2 × 10^−2^ atm) conditions. Dry gas was obtained by circulating through P_2_O_5_. The wet gas was obtained by bubbling through saturated solution of KBr (*p*H_2_O = 2 × 10^−2^ atm). 

## 3. Results

### 3.1. X-ray, SEM, and EDS Characterization

**Figure 2:**
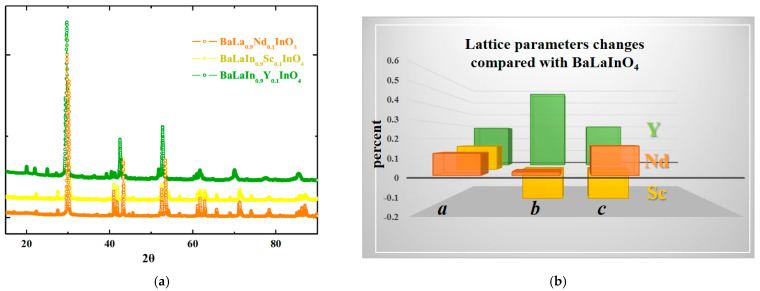
The XRD-patterns of isovalent-doped compositions BaLa_0.9_Nd_0.1_InO_4_, BaLaIn_0.9_Sc_0.1_O_4_, BaLaIn_0.9_Y_0.1_O_4_ (**a**) and lattice parameters changes of these compositions compared with BaLaInO_4_ (**b**).

**Figure 3:**
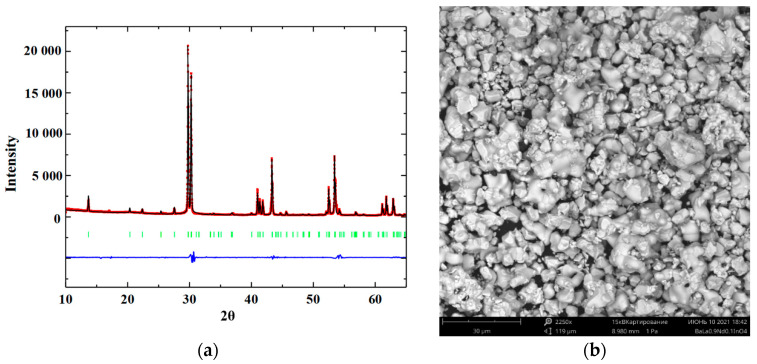
The refinement of XRD-pattern (**a**) and SEM-images of the compositions BaLa_0.9_Nd_0.1_InO_4_ (**b**).

XRD-analysis of powder sample BaLa_0.9_Nd_0.1_InO_4_ confirmed the single-phase nature of obtained composition. Both previously obtained and investigated scandium-doped BaLaIn_0.9_Sc_0.1_O_4_ and yttrium-doped BaLaIn_0.9_Y_0.1_O_4_ compositions, and the neodymium-doped sample crystallize in the *Pbca* space group (Figure 2a) and isostructural to the matrix composition BaLaInO_4_. The results of Rietveld refinement of BaLa_0.9_Nd_0.1_InO_4_ composition are presented in the Figure 3a and Table 1 The changes in lattice parameters of these compositions during doping compared with BaLaInO_4_ (*a* = 12.932(3) Å, *b* = 5.906(0) Å, *c* = 5.894(2) Å) are presented in the Figure 2b and Table 2. As can be seen, despite of “plus” or “minus” difference in the ionic radii of the metals ($r_{{\mathrm{In}}^{3+}}$ = 0.80 Å, $r_{{\mathrm{Sc}}^{3+}}\;$= 0.745 Å, $r_{Y^{3+}}$ = 0.90 Å, $r_{{\mathrm{La}}^{3+}}$ = 1.216 Å, $r_{{\mathrm{Nd}}^{3+}}$ = 1.163 Å [63]), doping led to an increase in the *a* lattice parameter (interlayer space) for all compositions. At first sight, increase in the lattice parameter during doping by the ions with smaller ionic radii (Nd^3+^→La^3+^ and Sc^3+^→In^3+^) can be considered as contradiction. However, this is seeming contradiction, which can be explained on closer examination. 

It is obvious that doping led to the formation substitution defects M_La_ and M_In_. From the quasi-chemical point of view, they are neutral and can be written as ${\mathrm{Nd}}_{\mathrm{La}}^{\times }$, ${\mathrm{Sc}}_{\mathrm{In}}^{\times }$, and $Y_{\mathrm{In}}^{\times }$. On the contrary, from the crystallochemical point of view, these defects can be written as ${\mathrm{Nd}}_{\mathrm{La}}^{\delta -}$, ${\mathrm{Sc}}_{\mathrm{In}}^{\delta +}$, and $Y_{\mathrm{In}}^{\delta +}$ because of difference in the electronegativity of elements ($\chi_{\mathrm{In}}$ = 1.78, $\chi_{\mathrm{Sc}}$ = 1.36, $\chi_{Y}$ = 1.22, $\chi_{\mathrm{La}}$ = 1.10, $\chi_{\mathrm{Nd}}$ = 1.14 [64]). In other words, the redistribution of the electron density and the change in the effective charges on the atoms take place, which leads to changes in the energy and length of metal-oxygen bonds. Accordingly, the change in the lattice parameters during doping may not have a direct correlation with the size of the dopant. We can conclude that observing one-way trend of increase in *a* lattice parameter is due to the appearance of additional repulsion effects of different nature ions in one sublattice. It should be noted that in contrast to the perovskite 3D structure where the octahedrons are connected by all six vertices, the layered perovskite structure contains the octahedra layers bonded only by axial oxygens and non-bonded by apical oxygens. Thus, this structure is more flexible and more easily able to change the crystal-chemical distances in comparison with the classic perovskite structure. 

Verification of chemical composition of obtained sample BaLa_0.9_Nd_0.1_InO_4_ was performed using SEM coupled with energy-dispersive diffraction analysis. Good agreement between theoretical and obtained values is observed (Table 3). Sample consists of irregularly rounded grains ~3–5 µm, forming agglomerates up to 15 µm (Figure 3). 

### 3.2. TG-Measurements

**Figure 4:**
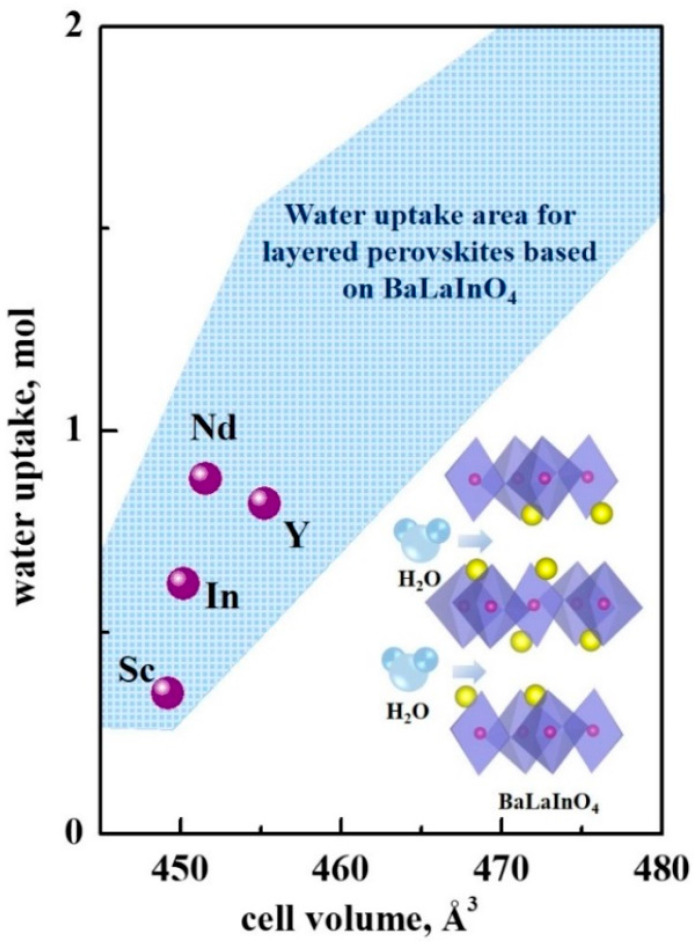
The dependency of water uptake vs. unit cell volume for the compositions BaLa_0.9_Nd_0.1_InO_4_ (Nd), BaLaIn_0.9_Sc_0.1_O_4_ (Sc) [54], BaLaIn_0.9_Y_0.1_O_4_ (Y) [55], BaLaInO_4_ (In) [49]. The blue area defines the water uptake area for the acceptor- and donor-doped compositions based on BaLaInO_4_ with 0.1 mol dopant content [62]. Schematic illustration of water insertion into BaLaInO_4_ structure is also shown.

For the materials with classic perovskite structure ABO_3–δ_, the possibility of water uptake depends on the amount of oxygen vacancies in the structure and is determined by the value of δ in the general case. However, for the layered perovskite AA′BO_4_, this process is provided by the intercalation of OH-groups into the space between perovskite layers (inset in the Figure 4):

(1) $$H_{2}O+O_{O}^{x}↔{(\mathrm{OH})}_{O}^{•}+{(\mathrm{OH}\;)}_{i}^{\prime }$$

Moreover, the amount of water uptake for heterovalent-doped samples based on BaLaInO_4_ does not depend on the concentration of oxygen vacancies, but it is determined by the unit cell volume of the composition [62]. The water uptake area for the acceptor- and donor-doped compositions based on BaLaInO_4_ is presented in the Figure 4 in blue color. As can be seen, the water uptake for the isovalent-doped compositions BaLa_0.9_Nd_0.1_InO_4_, BaLaIn_0.9_Sc_0.1_O_4_, and BaLaIn_0.9_Y_0.1_O_4_ is well correlated with the value of the unit cell volume also. Thus, we can conclude that the doping mechanism does not affect the possibility of water uptake for compositions based on BaLaInO_4_. The only factor determining the amount of water absorption is the unit cell volume of the layered perovskite.

**Figure 5:**
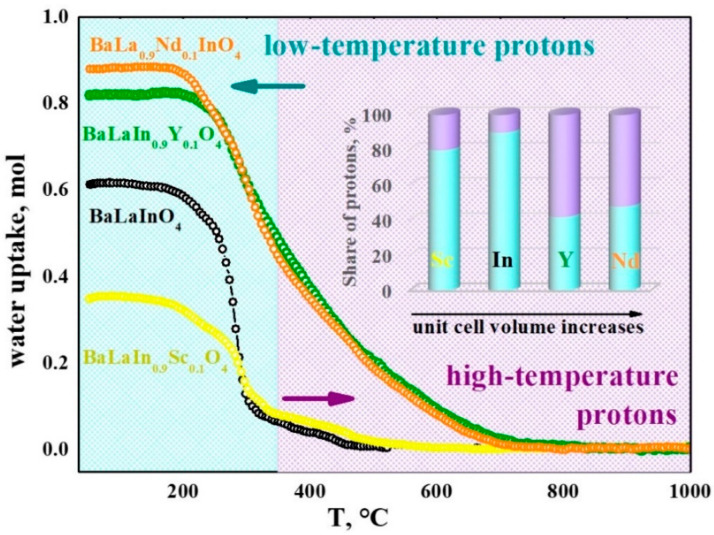
The TG-data for the compositions BaLa_0.9_Nd_0.1_InO_4_, BaLaIn_0.9_Sc_0.1_O_4_ [54], BaLaIn_0.9_ Y_0.1_O_4_ [55], BaLaInO_4_ [49]. The shares of protons with different thermal stabilities for the doped compositions are also shown.

The TG-curve for the BaLa_0.9_Nd_0.1_InO_4_ composition in comparison with curves for previously reported undoped and scandium- and yttrium-doped compositions are presented in the Figure 5. All samples are dehydrated in several steps. The appearance of several steps on the TG-curve confirms the presence in the structure of non-equivalent protons characterized by different thermal stabilities. The dehydration temperature is higher when OH-groups are more strongly bonded to the crystal lattice and are less involved in hydrogen bonds. Low-temperature protons (200–350 °C) are removed first and corresponded to the strongly bonded hydroxyl groups [55]. High-temperature protons (350–700 °C) are removed later and corresponded to the weakly bonded or relatively isolated hydroxyl groups. As can be seen (inset in the Figure 5), the share of low-temperature protons decreases with the increase in the unit cell volume of doped compositions. 

### 3.3. Electrical Properties

**Figure 6:**
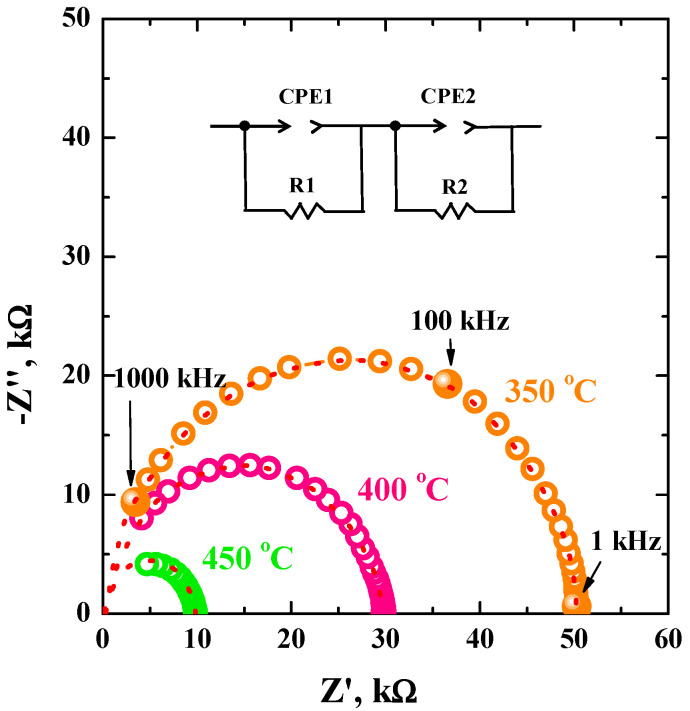
The Nyquist plots obtained at 350, 400, and 450 °C under wet air for the composition BaLa_0.9_Nd_0.1_InO_4_.

The impedance spectroscopy method was used for the investigation of electrical properties. The Nyquist plots for BaLa_0.9_Nd_0.1_InO_4_ composition are presented in the Figure 6. Fitting of experimental data (red line) was made. The applied equivalent circuit is presented in the Figure 6, where R_1_ is the bulk resistance, R_2_ is the grain boundaries resistance. The bulk resistance values R_1_ were used for the calculation of electrical conductivity (Table 4).

**Figure 7:**
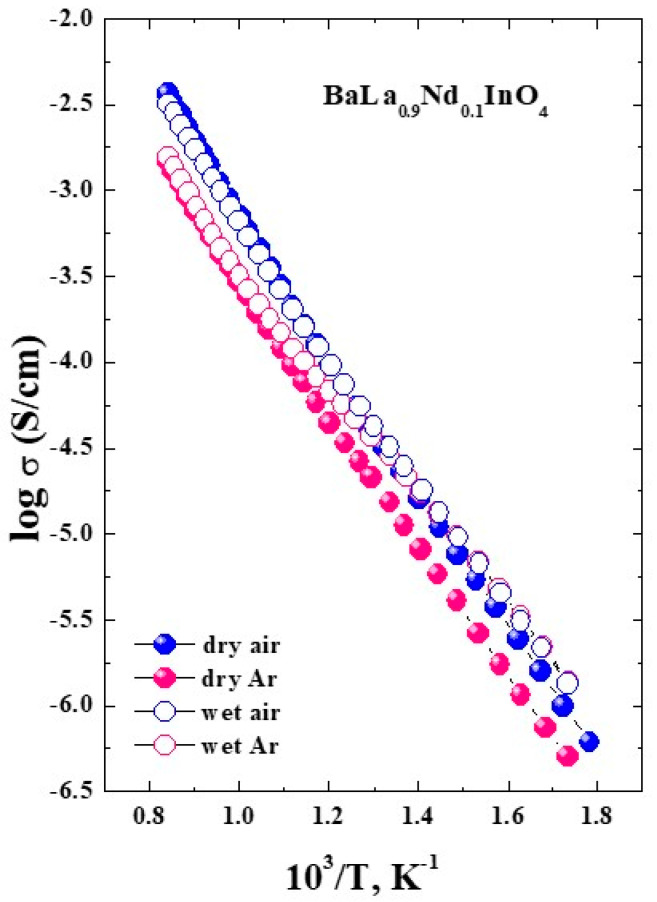
The conductivity dependencies form temperature for BaLa_0.9_Nd_0.1_InO_4_ composition.

Figure 7 represents the temperature dependencies of conductivities for BaLa_0.9_Nd_0.1_InO_4_ composition. The conductivity obtained at dry Ar (filled red symbols) is lower than value obtained under dry air (filled blue symbols) in whole investigated temperature range. As it was shown earlier for the undoped and doped materials based on BaLaInO_4_, the conductivity in dry Ar (~10^–5^ atm) is conductivity of oxygen-ionic conductivity [51,52,54]. Thus, we can say that the composition BaLa_0.9_Nd_0.1_InO_4_ is characterized by the mixed oxygen ionic-hole conductivity in dry air. The oxygen-ionic transport numbers can be calculated as:

(2) $$t_{O^{2-}}=\frac{\sigma_{O^{2-}}}{\begin{array}{c} \sigma_{\mathrm{tot}} \end{array}}=\frac{\sigma_{\mathrm{Ar}}}{\sigma_{\begin{array}{c} \begin{array}{c} \mathrm{air} \end{array} \end{array}}}$$

and they are about 40% in the whole temperature range. At the same time, the $t_{O^{2-}}$ for the undoped composition is about 20% [49] and they are also independent of the temperature. Thus, doping leads to the increase in total conductivity and share of oxygen-ionic conductivity. 

The relation of conductivity at wet air (open blue symbols) and at wet Ar (open red symbols) is different in comparison with dry condition. At high temperatures (higher than 450 °C), the conductivity values in wet air are higher compared to values in wet Ar. At low temperatures (below 450 °C), the conductivity in wet air is comparable with the conductivity in wet Ar. Because proton concentration becomes significant at low temperatures, the increase in conductivity in wet Ar compared to dry Ar is due to the formation of protonic species which leads to a decrease in the contribution of the whole conductivity:

(3) h•+12H2O +Oi″↔14O2+(OH)i′

The protonic conductivity was calculated as the difference between conductivity in wet and dry Ar. The proton transport numbers t_p_ were calculated according to the equation:

(4) $$t_{p}=\frac{\sigma_{\mathrm{wet}\;\mathrm{Ar}}-\sigma_{\mathrm{dry}\;\mathrm{Ar}}}{\sigma_{\mathrm{wet}}}$$

They were about 95% below 400 °C for the composition BaLa_0.9_Nd_0.1_InO_4_. The same protonic conductivity transport numbers were obtained earlier for the BaLaIn_0.9_Sc_0.1_O_4_ [54] and BaLaIn_0.9_Y_0.1_O_4_ [55] compositions also. 

The protons mobility values were calculated according to the formula:

(5) $$\mu_{H^{+}}=\frac{\sigma_{H^{+}}}{e\cdot c_{H^{+}}}$$

**Figure 8:**
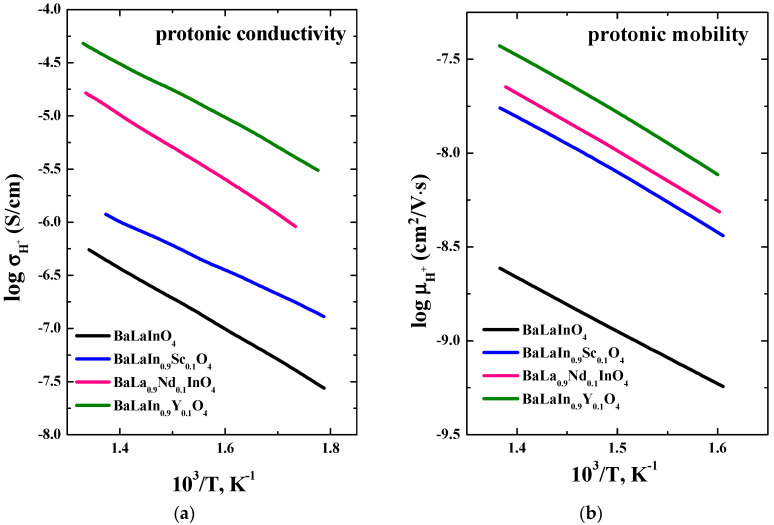
Dependencies of protonic conductivity (**a**) and mobility (**b**) from temperature for the compositions BaLa_0.9_Nd_0.1_InO_4_, BaLaIn_0.9_Sc_0.1_O_4_ [54], BaLaIn_0.9_Y_0.1_O_4_ [55], BaLaInO_4_ [49].

**Figure 9:**
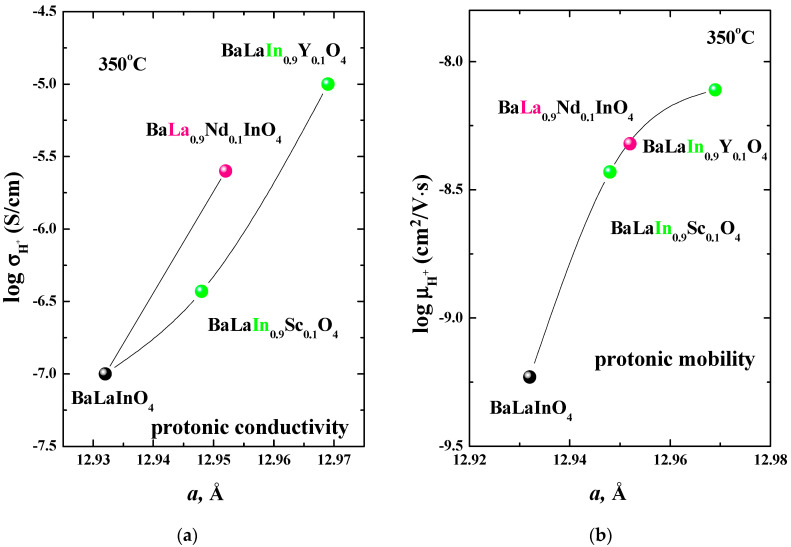
Dependencies of protonic conductivity (**a**) and mobility (**b**) from *a* lattice parameter for the compositions BaLa_0.9_Nd_0.1_InO_4_, BaLaIn_0.9_Sc_0.1_O_4_ [54], BaLaIn_0.9_Y_0.1_O_4_ [55], BaLaInO_4_ [49] at 350 °C.

The temperature dependencies of proton conductivity and mobility for the BaLa_0.9_Nd_0.1_InO_4_ composition in comparison with curves for previously reported undoped, scandium-doped, and yttrium-doped compositions are presented in the Figure 8a,b correspondingly. The isovalent doping leads not only to an increase in the protonic conductivity but also proton mobility. Figure 9 demonstrates protonic conductivity and mobility vs. *a* lattice parameter for the undoped BaLaInO_4_ and isovalent-doped compositions BaLa_0.9_Nd_0.1_InO_4_, BaLaIn_0.9_Sc_0.1_O_4_, and BaLaIn_0.9_Y_0.1_O_4_ at 350 °C. Both conductivity and mobility of protons increase with an increasing in *a* lattice parameter for all isovalent-doped compositions. In the other words, the increase in protonic conductivity is provided not only by the changes in the concentration of protons, but also by an increase in their mobility. It is obvious that this increase in mobility is due to the expansion of the interlayer space (*a* lattice parameter) which facilitates the transport of protons. 

**Figure 10:**
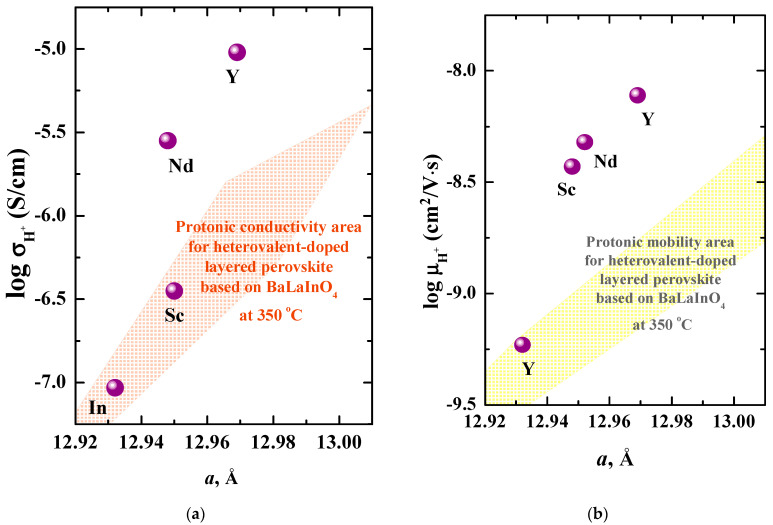
Dependencies of protonic conductivity (**a**) and mobility (**b**) from *a* lattice parameter for the compositions BaLa_0.9_Nd_0.1_InO_4_ (Nd), BaLaIn_0.9_Sc_0.1_O_4_ (Sc) [54], BaLaIn_0.9_Y_0.1_O_4_ (Y) [55], BaLaInO_4_ (In) [49] at 350 °C. The orange and yellow areas are defined as the protonic conductivity and mobility areas correspondingly for the acceptor- and donor-doped compositions based on BaLaInO_4_ with 0.1 mol dopant content [62].

Figure 10 represents the same dependencies of protonic conductivity and mobility vs. *a* lattice parameter for isovalent-doped compositions in comparison with heterovalent-doped compositions with the same (0.1 mol) dopant content (colored areas in the Figure 10). As can be seen, the isovalent doping allows to increase more significantly the conductivity and mobility of protons in comparison with heterovalent doping at the same dopant concentration. In the other words, the mobility of protons in the heterovalent-doped compositions is lower in comparison with isovalent-doped compositions at the same value of *a* lattice parameter. Thus, there is an additional factor affecting the mobility of protons in the layered perovskites. As it was shown for the heterovalent-doped compositions [62], the mobility of protons decreased at “high” (more ~ 0.1 mol) dopant concentrations due to the formation of proton-aggregating clusters:

(6) $$M_{A}^{\prime }+{(\mathrm{OH})}_{o}^{•}\to {\left(M_{A}^{\prime }\cdot {(\mathrm{OH})}_{o}^{•}\right)}^{\times }$$

(7) $$M_{B}^{•}+{(\mathrm{OH})}_{i}^{\prime }\to {\left(M_{B}^{•}\cdot {(\mathrm{OH})}_{i}^{\prime }\right)}^{\times }$$

It is obvious that the process of cluster formation occurs at lower concentrations of the dopant also. Opposite to the heterovalent doping, the isovalent doping can lead to the indirect formation of partially charged defects (${\mathrm{Nd}}_{\mathrm{La}}^{\delta -}$, ${\mathrm{Sc}}_{\mathrm{In}}^{\delta +}$, $Y_{\mathrm{In}}^{\delta +}$) only. Accordingly, the share of proton-containing clusters is significantly lower or even absent. This suggests that isovalent doping of layered perovskites AA′BO_4_ is a more promising method for improving transport properties compared with heterovalent doping. This method can be applied for obtaining novel advanced proton-conducting ceramics which can be used as electrolytic material in different energy conversion devices. 

## 4. Conclusions

In this paper, neodymium-doped phase based on layered perovskite BaLaInO_4_ was obtained for the first time. The ability for water intercalation and proton transport was proved. It was shown that the composition BaLa_0.9_Nd_0.1_InO_4_ is the predominant proton conductor in the wet air below 400 °C. The comparative analysis of transport properties of isovalent-doped and heterovalent-doped protonic conductors based on BaLaInO_4_ was also carried out. First, all types of doping leads to an increase in the protonic conductivity values. Second, the increase in the protonic conductivity is provided not only by an increase in the concentration of protons, but also by an increase in their mobility. The increase in mobility is due to the expansion of the interlayer space which facilitates the transport of protons. Third, the isovalent doping allows to increase more significantly the conductivity and mobility of protons in comparison with heterovalent doping at the same dopant concentration. Isovalent doping of layered perovskites AA′BO_4_ is the promising method for improving transport properties and obtaining novel advanced proton-conducting ceramic materials. 

## Figures and Tables

**Table 1:** Refined structural parameters from XRD-data.

Atom	Site	x	y	z
Ba	8c	0.1439(2)	−0.0028(3)	0.0031(1)
La	8c	0.1439(2)	−0.0028(3)	0.0031(1)
In	4b	0.5	0	0
O(1)	8c	0.0193(1)	0.203(3)	0.203(3)
O(2)	8c	0.332(4)	0.0193(1	0.0095(4)

R_p_ = 4.22, R_wp_ = 3.31, χ^2^ = 1.78.

**Table 2:** The parameters of unit cell for the isovalent-doped compositions based on BaLaInO_4_.

Composition	BaLa_0.9_Nd_0.1_InO_4_	BaLaIn_0.9_Sc_0.1_O_4_ [54]	BaLaIn_0.9_Y_0.1_O_4_ [55]
*a*, Å	12.948(5)	12.951(9)	12.969(1)
*b*, Å	5.907(2)	5.895(1)	5.883(2)
*c*, Å	5.903(5)	5.883(2)	5.911(6)
V, Å^3^	451.55(7)	449.19(8)	455.24(7)

**Table 3:** The average element ratios determined by EDS analysis for the sample BaLa_0.9_Nd_0.1_InO_4_ (theoretical values are in brackets).

Element	Ba	La	Nd	In
Content, atomic %	33.6 (33.3)	29.7 (30.0)	3.2 (3.3)	33.5 (33.4)

**Table 4:** The Nyquist plots fitting results.

Element	Value (450 °C)	Value (400 °C)	Value (350 °C)
CPE1, F	2.3 × 10^−12^	2.1 × 10^−12^	3.1 × 10^−12^
R1, kΩ	9	27	46
CPE2, F	4.2 × 10^−10^	3.8 × 10^−10^	2.2 × 10^−10^
R2, kΩ	1	3	4

## Data Availability

Not applicable.
